# PSYCHOMETRIC PROPERTIES OF FUNCTIONAL CAPACITY TESTS IN CHILDREN AND ADOLESCENTS: SYSTEMATIC REVIEW

**DOI:** 10.1590/1984-0462/;2018;36;4;00002

**Published:** 2018

**Authors:** Janaina Cristina Scalco, Renata Martins, Patricia Morgana Rentz Keil, Anamaria Fleig Mayer, Camila Isabel Santos Schivinski

**Affiliations:** a Universidade do Estado de Santa Catarina, Florianópolis, SC, Brasil.

**Keywords:** Child, Exercise tolerance, Validity of tests, Reproducibility of results, Criança, Tolerância ao exercício, Validade dos testes, Reprodutibilidade dos testes

## Abstract

**Objectives:**

To identify studies that evaluated psychometric properties of functional capacity tests in children and adolescents, and to verify which of these have satisfactory properties of measurement.

**Data sources:**

Searches on MEDical Literature Analysis and Retrieval System Online (MEDLINE), Cumulative Index to Nursing and Allied Health Literature (CINAHL) and Scientific Electronic Library Online (SciELO) databases without limiting period or language. Two investigators independently selected articles based on the following inclusion criteria: children and/or adolescent population (healthy or with cardiorespiratory diseases); and assessment of psychometric properties of functional capacity tests. Studies with (I) adult samples, (II) sample with neurological diseases, and (III) on reference values or prediction equations only were excluded.

**Data synthesis:**

From the total of 677 articles identified, 11 were selected. These evaluated the psychometric properties of the following tests: 6-minute walk test (6MWT) (n=7); 6MWT and the 3-minute step test (3MST) (n=1); and Incremental Shuttle Walk Test (ISWT) (n=3). Reproducibility and reliability were good for 6MWT and ISWT, and moderate for 3MST. The ISWT showed high validity measures for both healthy children and children with chronic respiratory disease. The validity of 6MWT varied across studies, and should be analyzed according to the health conditions of test takers. The validity of 3MST is unclear, and further studies in pediatric population are required.

**Conclusions:**

Most studies investigated 6MWT measurement properties. Validity of 6MWT varied according to different pediatric populations. The use of 6MWT, ISWT and 3MST tests to measure clinically important changes in children and adolescents with cardiorespiratory diseases is still unclear.

## RESUMO

**Objetivos:**

Identificar estudos que avaliam as propriedades psicométricas dos principais testes de capacidade funcional utilizados em crianças e adolescentes e verificar os testes que apresentam propriedades de mensuração satisfatórias.

**Fontes de dados:**

Pesquisa nas bases de dados *MEDical Literature Analysis and Retrieval System Online* (MEDLINE), *Cumulative Index to Nursing and Allied Health Literature* (CINAHL) e *Scientific Electronic Library Online* (SciELO), sem limitar o período ou idiomas. Dois investigadores selecionaram de forma independente os artigos com base nos critérios de inclusão: estudos que avaliaram propriedades psicométricas dos testes de capacidade funcional em crianças e/ou adolescentes saudáveis ou com doenças cardiorrespiratórias. Foram excluídas pesquisas que incluíram indivíduos adultos e/ou participantes com doenças neurológicas e estudos que exclusivamente estabeleceram valores de referência ou equações de predição.

**Síntese dos dados:**

Do total de 677 artigos, foram selecionados 11 compatíveis com o tema; destes, 7 avaliaram propriedades psicométricas do teste de caminhada de 6 minutos (TC6), 1 avaliou tanto o TC6 quanto o teste do degrau de 3 minutos (TD3), e 3 avaliaram o *teste incremental Shuttle Walk Test* (ISWT). As evidências sobre as propriedades de reprodutibilidade e confiabilidade são boas para o TC6 e ISWT e moderadas para o TD3. O ISWT mostra medidas de validade altas para crianças saudáveis e para aquelas com doença respiratória crônica. As medidas de validade do TC6 variam entre as populações estudadas e devem ser consideradas de maneira particular para cada condição de doença. A validade do TD3 ainda deve ser esclarecida na população pediátrica.

**Conclusões:**

O TC6 apresenta maior número de investigações sobre suas propriedades, mas sua validade apresenta variabilidade para diferentes populações pediátricas. A capacidade dos testes TC6, ISWT e TD3 de mensurar mudanças clinicamente importantes em crianças e adolescentes com doenças cardiorrespiratórias é desconhecida.

## INTRODUCTION

Keeping an active lifestyle, by practicing sports and participating in games, is essential for the normal development of a child^1^ - and it has been already established that regular physical activity provides quality of life and benefits to the overall state of health to healthy children or children diagnosed with chronic diseases.^2^ ^,^ ^3^ However, individuals with pulmonary diseases may lose exercise capacity and face consequent limitations in functional activities.^2^ ^,^ ^4^

Individual response to exercise is an important instrument for clinical evaluation, as integrated responses of the respiratory, cardiac, metabolic and muscular systems are obtained.^5^ Several tests are aimed to evaluate human response to exercise and, nowadays, the incremental cardiopulmonary exercise testing (CPET) is considered the gold standard to assess maximum exercise capacity, although it demands high-cost equipment and specialized professionals.^5^

On the other hand, submaximal exercise tests have been used to assess functional capacity and reflect one’s maximum capacity to perform daily life activities (DLA), which are mostly submaximal ones.^6^ Among functional capacity tests, the 6-minute walk test (6MWT) is the most well-known and capable of pointing out the limitations of individuals to perform DLAs^6^ ^,^ ^7^ even in the pediatric population.^5^ ^,^ ^8^

To evaluate children and adolescents, the indication is a test that can effectively evaluate what it proposes to in addition to being clinically applicable and promoting reliable results. The instrument must, therefore, have satisfactory psychometric properties,^9^ an important feature to detect the minor effects of a treatment.^10^

Thus, this systematic literature review that aimed to identify studies on the psychometric properties of the main functional capacity tests applied to children and adolescents allows to identify tests that have qualified measurement properties, enabling its indication and use in clinical practice.

## METHOD

In order to develop and expose this review, the recommendations for the presentation of systematic reviews of the Preferred Reporting Items for Systematic Reviews and Meta Analysis (PRISMA) were considered. Then, a systematic search of the literature was carried out in April 2017 on the Literature Analysis and Retrieval System Online (MEDLINE), via OVID MEDLINE, and on the Cumulative Index to Nursing and Allied Health Literature (CINAHL), via Elton B. Stephens Company (EBSCO), and the Scientific Electronic Library Online (SciELO). Original search strategies were created for the first two databases, and they are listed in Chart 1. On SciELO, the following combination of descriptors was used: “criança” *and* “teste de exercício” and their English equivalent “*children*” *and* “*exercise test*”. The search was not limited by other filters such as language or date of publication.

**Chart 1:** Search strategy.

CINAHL with Full Text (EBSCO)	
1. “Pediatr*”	9. “Exercise capacity”
2. “Child*”	10. “Activity of daily living”
3. “Adolescent”	11. (MH “Functional status”)
4. “School age”	12. “Physical capacity”
5. (MH “Child, Preschool”)	13. “Functional capacity”
6. (MH “Child”) AND (1 OR 2 OR 3 OR 4 OR 5)	14. “Everyday activities”
7. (MH “Exercise test”)	15. (“Every day activities”) AND (7 OR 8 OR 9 OR 10 OR 11 OR 12 OR 13 OR 14)
8. “Exercise tolerance”	16. 15 AND 6
MEDLINE via OVID	
1. Randomized controlled trials as Topic/	25. School age.mp.
2. Randomized controlled trial/	26. Child, Preschool/
3. Random allocation/	27. 22 or 23 or 24 or 25 or 26
4. Double blind method/	28. Step test.mp.
5. Single blind method/	29. Shuttle walk test.mp.
6. Clinical trial/	30. Six-minute walk test.mp.
7. exp Clinical Trials as Topic/	31. Cardiopulmonary test.mp.
8. (clinic$ adj trial$1).tw.	32. Ergoespirometry.mp.
9. 1 or 2 or 3 or 4 or 5 or 6 or 7 or 8	33. Free running test.mp.
10. (Follow up adj (study or studies).tw.	34. Exercise Test/
11. (observational adj (study or studies).tw.	35. Exercise capacity.mp.
12. Longitudinal.tw.	36. Functional capacity.mp.
13. Retrospective.tw.	37. Functional status.mp.
14. review.pt.	38. Physical capacity.mp.
15. 10 or 11 or 12 or 13 or 14	39. 28 or 29 or 30 or 31 or 32 or 33 or 34 or 35 or 36 or 37 or 38
16. 9 or 15	40. lResponsiveness.mp.
17. Case report.tw.	41. Minimal clinically important difference.mp.
18. Letter/	42. Equation reference.mp.
19. Historical article/	43. Reference Values/
20. 17 or 18 or 19	44. Reliability.mp.
21. 16 not 20	45. Validity.mp.
22. Child*.mp.	46. Reproducibility.mp.
23. Pediatr*.mp.	47. 40 or 41 or 42 or 43 or 44 or 45 or 46
24. Adolescent*.mp.	48. 21 and 27 and 39 and 47

CINAHL: Cumulative Index to Nursing and Allied Health Literature; MEDLINE: MEDical Literature Analysis and Retrieval System Online; EBSCO: Elton B. Stephens Company.

The following inclusion criteria were considered:

1. studies whose purpose was to evaluate some psychometric properties (validity, reliability, reproducibility, responsiveness, minimal clinically important difference) of functional capacity tests;
2. tests evaluated in healthy children and/or adolescents (up to 19 years old, according to WHO classification)^11^ or with cardiorespiratory diseases.

The surveys involving adult samples or whose participants had associated neurological diseases were excluded. Also, studies that established exclusively reference values or prediction equations were not included in this review, but these terms were included in the search strategy because some studies evaluated psychometric properties of the tests simultaneously.

Two independent researchers performed the screening of studies by analyzing all of them and respecting the pre-established inclusion and exclusion criteria. Initially, the headings were assessed and, when compatible, articles were selected for abstract evaluation. After analyzing abstracts chosen consensually, the articles were obtained in full and read for confirmation of compatibility of the content with the criteria required for this review. Divergence as to exclusion of a heading, abstract, or full text was discussed by researchers until consensus. To ensure the inclusion of all relevant publications, the reference lists of all studies selected were also searched manually by the evaluators.

The checklist Strengthening the Reporting of Observational Studies in Epidemiology (STROBE), which encompasses recommendations to improve the methodological quality of observational studies,^12^ was adapted with scores to characterize studies. The checklist is composed of 14 items stratified or not in subitems, totaling 22 items. Each item was assigned a proportional score, with maximum sum of 20 points.

The psychometric properties of each test were classified as “good”, “moderate”, “poor”, and “unknown”. Validity and reliability/reproducibility were considered “good” when most studies had a significant correlation ≥0.75 or significant p-value, “moderate” when between 0.40 and 0.75, and “poor” when <0.40.^13^ Regarding other populations, the tests applied to more than two populations were considered “good”; to two populations, “moderate”; and to one population only, “bad”. Some of the psychometric properties were not evaluated in the studies selected, to which the “unknown” classification was attributed.

## RESULTS

**Figure 1:**
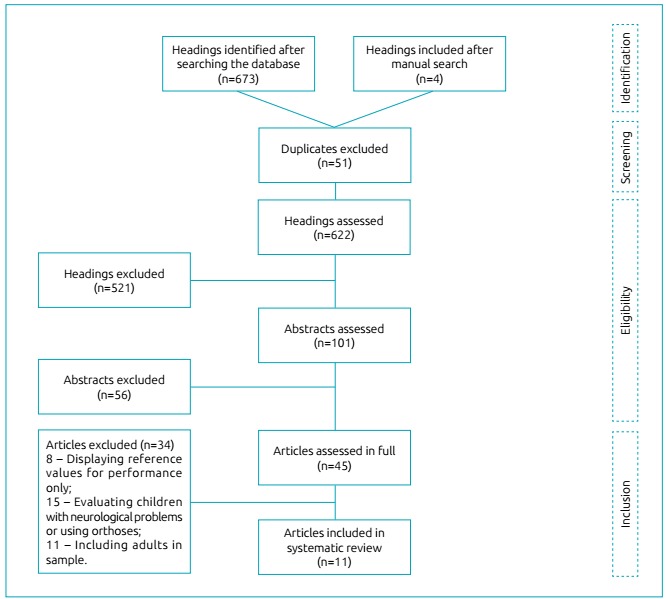
Flow chart of studies’ selection.

In total, 677 articles were identified in database and manual searches. After exclusion of duplicates, 622 were sent for peer selection of headings. Of these, 101 were considered eligible for selection of abstracts and 45 for final analysis, that is, full reading of the article. Passed these phases, 11 articles were included in this review. Article selection and exclusion stages are shown in Figure 1.

Most articles selected (seven) evaluated the psychometric properties of the 6MWT; one article evaluated both the 6MWT and the 3-minute step test (3MST), while three evaluated the Incremental Shuttle Walk Test (ISWT), or its adapted version Modified Shuttle Walk Test (MSWT). These studies are listed in Charts 2 and 3.

**Chart 2:** Description of studies evaluating the psychometric properties of the field test (6MWT).

Author/ year	*Checklist* STROBE	Population and sample	Method	Psychometric property assessed	
Gulmans et al*.*, 1996	15.1	Children and adolescents with CF aged 8 to 18 years (mean 11.1±2.2 years) (n=15 validity) (mean 14.5±2.0 years) (n=23 reproducibility)	V: 1 6MWT and a test in cycle (10W increment if height was <160 cm, or FEV1 <60%, or 15 W per minute) performed for at least two days before or two days after 6MWT. R: 2 6MWT (8-m lane, encouraging every 16 m) in the same day and repeated after a week.	Validity Reproducibility	Correlation between CD and VO_2máx_ (r=0.76). r=0.90.
Li et al*.*, 2005	16.1	Healthy Chinese children aged 12 to 16 years (mean 14.2±1.2 years) (n=74 validity) (n=52 reliability)	V concurrent: maximum CPET on treadmill and 6MWT with interval of up to 2 weeks between them. Re: 6MWT was repeated at intervals of 2 to 4 weeks.	Validity Reliability	Correlation between DC. 6MWT and VO_2max_ (r=0.44). ICC=0.94.
Lammers et al*.*, 2011	14.1	Children with pulmonary hypertension aged 6 to 18 years (mean 13.0±3.0 years)	V: All of them performed maximum CPET on cycle ergometer and the 6MWT.	Validity	Correlation between DC. 6MWT and VO_2peak_ with VO_2VT_ (r=0.49 e r=0.40. respectively)
Cunha et al*.*, 2006	12.1	Children with CF aged 8 to 14 years (mean 11.0±1.9 years) (n=16)	Two 6MWT (28-m lane) were performed on the same day, with a minimum interval of 30 min between them.	Reproducibility	No difference between DCs (p=0.31). which shows good reproducibility
Priesnitz et al*.*, 2009	15.1	Healthy children and adolescents aged 6 to 12 years (mean 11.7 years)	R: Two 6MWT (30-m lane), with interval of 30 min	Reliability	ICC: 0.74.
Morinder et al*.*, 2009	14.1	Obese children and adolescents aged 8 to 16 years (mean 13.2 years) (n=49 reproducibility) (n=250 validity)	V: 6MWT and a submaximal exercise test on a stationary bicycle for same-day comparison. R: Two 6MWT (70-m lane), with mean interval of 4 days	Validity Reproducibility	Correlation between DC in 6MWT with VO_2max_ (r=0.34). ICC=0.84.
Mandrusiak et al*.*, 2009*	13.9	Children and adolescents with CF aged 7 to 17 years (mean 13.1±2.7 years) hospitalized for respiratory exacerbation (n=18)	Re: After one or two days of hospital admission, a 6MWT was performed per day on two consecutive days.	Reliability	ICC=0.93

*Check-list* STROBE: score of methodological characteristics of studies (maximum sum of 20 points); CF: cystic fibrosis; n: sample number; V: validity; FEV1: first-second forced expiratory volume; R: reproducibility; Re: reliability; 6MWT: 6-minute walk test; W: watt; CPET: cardiopulmonary test; DC: distance covered; VO_2_: oxygen consumption; max: maximum; min.: minutes; m: meters; ICC: intraclass correlation coefficient; VO_2VT_: oxygen consumption at ventilatory threshold.

**Chart 3:** Description of studies evaluating the psychometric properties of the field tests (6MWT, ISWT/MSWT, 3MST).

Author/ year	Checklist STROBE	Population and sample	Method	Psychometric property assessed	
Balfour-Lynn et al*.*, 1998	13.1	Children with symptomatic CF 6-18 years (mean 12.5 years) (n=54, validity) (n=12 reproducibility - 3MST) (n=9 reproducibility - 6MWT)	V: two 3MST performed and compared to two 6MWT (17-m lane), with interval of 30 min between them on the same day. Re: 3MST and 6MWT performed on two consecutive days. For all analyzes, we used the change of the SpO_2_ parameters, HR, degree of dyspnea.	Validity	3MST produced significantly higher HRs and Borg compared to the 6MWT. The decrease in SpO_2_ was similar between tests. Relation between SpO_2_ decrease and baseline FEV1 also similar in both tests (3MST r = 40.52 and 6MWT r = 40.51)
				Reproducibility	3MST: (SpO_2_: -2.1 to 2.5; HR: -38.0 a 34.0; Borg: -1.5 a 1.5) 6MWT: (SpO_2_: -1.7 to 1.0; HR:-34.0 a 39.0; Borg: -1.1 a 1.9).
Selvadurai et al*.*, 2003*	15.3	CF children aged 5-17 years (mean 6.8 years) n=35 (children aged 7 years or less, or too weak to perform a 20-m shuttle test run).	All children performed a CPET on a treadmill, two ISWT tests with simultaneous gas analysis and one ISWT test without oxygen mask in a maximum interval of one week.	Reproducibility	No significant difference between the two ISWT tests with the mask on or in comparison with and without mask for heart rate peak, DC, SpO_2_, Borg and VO_2peak_
				Validity	Strong correlation between DC and VO_2peak_ (r = 0.91); there were no significant differences in variables between ISWT and CPET.
Coelho et al*.*, 2007*	12.1	Children and adolescents with CF: CFG (n=14) and healthy: CG (n = 14) 7-15 years CFG (11.57 ± 2.50) CG (11.28 ± 1.85)	Each child performed at least two tests with a minimum 30-minute interval between them.	Reproducibility	CG: DC greater in the second test (p = 0.036). CFG: significant difference between first and second test only as to resting dyspnea scale, which increased in the second test, just like in healthy children (p = 0.042).
Lanza et al*.*, 2015	16.0	Brazilian Children and adolescents with normal pulmonary function and no chronic diseases (n=8) 6-18 years (mean age 12±2 years)	Two ISWT tests performed with interval of 30 min between them.	Reliability	ICC = 0.98 excellent reliability of distance covered between ISWT 1 and 2.

*Only part of the work was presented; checklist STROBE: composed of 14 items, each of which received scores with a maximum sum of 20 points; CF: cystic fibrosis; n: sample number; 3MST: 3-minute step test; 6MWT: six-minute walk test ; MSWT: Modified Shuttle Walk Test; m: meters ; min: minutes ; V: validity; R: reproducibility; Re: reliability; SpO_2_: peripheral oxygen saturation; HR: heart rate; CPT: cardiopulmonary test; ISWT: incremental shuttle walk test; DC: distance covered; VO_2_: oxygen consumption; ICC: intraclass correlation coefficient; CFG: cystic fibrosis group; CG: control group.

Chart 4 was elaborated from the results reported in selected studies, listing and classifying each psychometric property of the tests. It is noted that reliability and reproducibility are considered good for both 6MWT and ISWT. Also, minimal clinically important difference (MCID) and responsiveness were sorted as “unknown” for all tests.

**Chart 4:** Psychometric properties of functional capacity tests used in pediatrics.

Test	Validity	Reproducibility/Reliability	Feasibility	MCID	Other populations
6MWT	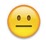	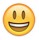	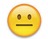	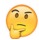	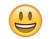
ISWT	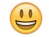	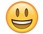	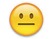	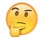	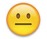
3MST	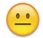	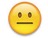	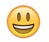	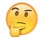	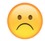

MCID: minimal clinically important difference; 6MWT: 6-minute walk test: 3 MST: 3-minute step test: ISWT: shuttle walk test; 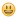: good; 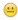: moderate; 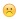: bad; 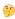: unknown.

## DISCUSSION

The analysis of cardiorespiratory response during exercise tests is an important tool to assess the impact of diseases and to monitor the effectiveness of interventions for individuals of all ages.^1^ ^,^ ^14^ However, the fact that, in addition to anthropometric differences, there are numerous physical variations between adults and children must not be lost sight of. Physiological aspects of children and adolescents are constantly changing; their systems are under development and maturation and may be influenced by genetic and ethnic factors, gender, physical activity, body composition, nutritional status, socioeconomic status, culture, climate, and geographic location.^15^ Thus, this population has a pattern (especially during growth spurt and puberty) that seems to interfere with their performance in tests and their responses during physical exercises.^16^ This justifies the need for more studies that evaluate and discuss the psychometric properties of functional capacity tests, specifically in pediatric populations.

Validity and reproducibility are related to the psychometric properties of the most investigated functional tests applied to the pediatric population. The validity of an instrument refers to its ability to analyze the phenomenon it intends to measure and indicates the extent to which its scores are an adequate reflection of the gold standard one. The reproducibility indicates the level of similarity between repeated measurements, reliability, and concordance parameters.^17^ ^,^ ^18^

The present review shows that, among functional capacity assessment tests, the 6MWT is the test of choice for most pediatric validation studies (healthy children and adolescents of different ethnicities, classified as obese, diagnosed with cystic fibrosis, pulmonary hypertension, and others), but important measures such as MCID have not been studied yet in pediatrics. This measure refers to the lowest relevant change in patients’ performance,^19^ which is representative of clinical improvement induced by pulmonary rehabilitation protocols or other interventions.^20^

Another matter that still raises doubts in validation studies is the possible relation of the distance covered in the 6MWT (DC_6MWT_) with measures representing the maximum capacity of exercise in different pediatric populations. Some studies have shown high or moderately high correlations between the 6MWT and the CPET,^22^ while others show weak correlations.^8^ ^,^ ^23^ It’s been confirmed that the 6MWT seems to reflect the maximum exercise capacity of children with moderate to severe cardiorespiratory diseases such as cystic fibrosis^21^ and hypertension^22^, but in obese^23^ and healthy children8, it reflects very little exercise capacity. Data presented by Lammers et al.^22^ reinforce these findings. Researchers point out a significant linear relationship between peak oxygen consumption (VO_2peak)_ and DC_6MWT_ only in children with pulmonary hypertension who walked less than 300 meters in the 6MWT. DC_6MWT_ represented 71% of the variation in VO_2peak_, but there was no association when the DC_6MWT_ was greater than 300 meters. As suggested by Bartels et al.,^24^ the response in the 6MWT seems to depend on both the population and the severity of the disease investigated. Thus, labeling the 6MWT as a maximal or submaximal measure is not justifiable before an adequate assessment of its validity in the target population, including mildly and severely affected patients.

The widespread use of 6MWT in both scientific and clinical practice is related to its simple, low-cost, easy-to-administer character,^6^ ^,^ ^7^ ^,^ ^25^ besides high levels of reproducibility and reliability^8^ ^,^ ^21^ ^,^ ^23^ ^,^ ^26^ ^,^ ^27^ ^,^ ^28^ and prediction equations and normality values already described for different ethnic groups.^26^ ^,^ ^29^ ^,^ ^30^ This is a continuous, self-paced walking test in which a constant speed is normally maintained,^31^ which may generate certain monotony for children upon its performance. This lack of motivation can interfere in performance and hinder accurate interpretation. Like the other tests accounted for in this review, the 6MWT was developed for the adult population eventually had its use diffused to the pediatric age group without changes in the administration protocol. This raises the debate about the need to develop (or adapt) tests with playful and motivational components in order to generate more interest and commitment by the children when performing them.

Externally paced tests such as 3MST and ISWT have the advantage of not depending solely on the patient’s motivation.^32^ ^,^ ^33^ In 3MST, children climb and descend a platform with a single step in a fixed time and frequency. Thus, its advantages are being fast, simple, portable and requiring little space for execution.^33^ Comparing 3MST and 6MWT in children with cystic fibrosis, 3MST seems to require more physiological adaptations to its execution. Balfour Lynn et al.^32^ reported a more significant increase in heart rate and Borg scale after 3MST, with no differences in peripheral oxygen desaturation. In the comparison between 3MST and CPET, even for children with moderate pulmonary disease, 3MST does not seem to detect important alterations, such as significant decreases in peripheral oxygen saturation during exercise.^34^ When evaluating the feasibility of 3MST applied to children who developed bronchiolitis obliterans after bone marrow transplantation, 3MST was shown to be an easy, well-tolerated and successfully performed test; in addition, it did not trigger hypoxemia and only one child took the maximum effort.^35^

There are several protocols for the step test with differences in run time (3, 4 and 6 minutes), in the cadence of climbs per minute (96/min, 30/min, 13/min, 15/min, 17/min), number of platform steps (1 or 2 steps), and size of steps.^32^ ^,^ ^35^ ^38^ The literature has not yet presented prediction equations regarding its performance nor values of normality for children and adolescents, which can hamper the comparison between studies and the identification of functional limitations upon clinical evaluation of pediatric patients.

In the walk test with incremental load, known as ISWT, the individual walks on and on a 10-meter track with progressive speed dictated by sound signals (increments of 0.17 m/s every minute) until no longer able to maintain the speed required.^31^ This protocol has been modified^39^ and an increase was applied to limit, from 12 to 15 speed levels (MSWT), in order to avoid the ceiling effect that the 12 speed levels could create in healthy or slightly-limited individuals, allowing patients to reach exhaustion.^40^ ^,^ ^41^ In pediatrics, the ISWT shows whether it is valid to evaluate functional and exercise capacity in children and adolescents with CF,^42^ which is highly related to the maximum oxygen volume (VO_2max_). Its reproducibility has been confirmed for this disease^42^ ^,^ ^43^ and in healthy children.^44^ When applied in asthmatics^37^ and in ex-premature infants, ISWT^45^ ^,^ ^46^ was shown sensitive to identify functional limitations compared to healthy controls. Recently, performance prediction equations (distance covered) for ISWT performed by Brazilian children and adolescents have been established,^44^ which facilitates applicability once the comparison with normal values ​​helps to identify functional limitations.

All three tests were found to involved only walking activity, which may restrict the evaluation of the influence of activities performed with the upper limbs on the limitation in ADL.^47^ Currently, researchers have discussed more comprehensive ways of assessing functional status of patients with lung diseases. In this regard, global tests, that is, including more than one task, seem to be the best choice.^48^ Hence, the Glittre-ADL multi-task test was developed. In addition to walking, it includes activities such as sitting on and standing up from a chair, walking up and down stairs, and moving objects with the upper limbs, being therefore considered more complete to evaluate the functional status of patients with pneumopathies.^47^ Its adaptation with playful components for application in the pediatric population (TGlitre P) is recent and has proved reproducible and acceptable for healthy children and adolescents.^49^

In the analysis of methodological quality, none of the articles reached the maximum score. That is, no research had all the recommended items for the best methodological quality of observational studies as indicated by STROBE. The studies covered on average 70% of recommended items. It is observed that a great part of the articles analyzed by this review did not score in the item “definition of sample calculation”; only items that, besides checking the psychometric properties, stipulated reference values ​​for the given test, scored. Note that the sample size of most chronic patient surveys was small, which, along with the lack of sampling methodology, does not allow to extrapolate the results to the reference population. Another item neglected by many studies was the “definition of preexisting hypotheses”, which reduced scores on STROBE. With regard to the analysis of “validity”, the absence of specific hypotheses about the expected correlations between variables makes it difficult to interpret the results and does not make it clear if they reflect the expected measure;^17^ nevertheless, we emphasize that all articles reviewed here considered at least 60% of recommendations for the best methodological quality.

When analyzing articles for this review, we found that the psychometric properties of 6MWT, 3MST and ISWT were also studied in groups of children and adolescents with cerebral palsy, cognitive disorders and Down’s syndrome.^36^ ^,^ ^50^ ^,^ ^51^ ^,^ ^52^ ^,^ ^53^ However, as these populations present other characteristics that impact the performance of tests, including level of motor function, cognitive level and use of orthoses, we decided not to discuss such studies and suggest that specific reviews on the applicability of these tests in children with motor disorders be created. Bartels et al.^24^ published a recent analysis of the measurement properties of the 6MWT in children with different chronic conditions (pulmonary, cardiac, neuromuscular, osteoarticular and other), which differs from all other by analyzing the psychometric properties of different functional capacity tests used to assess children and adolescents with cardiorespiratory diseases, aiming to assist professionals (clinician and/or researcher) in choosing the one that best suits their possibilities (physical space, materials) and which presents adequate psychometric measures to evaluate their target population. In addition, they indicate gaps in the literature that should be investigated, such as the absence of MCID for pediatric performance.

In summary, the 6MWT has been the most studied test applied to the pediatric population, but there are still divergences in results of validation studies and lack of studies investigating properties such as MCID. The ISWT has satisfactory psychometric properties and has been mostly studied in the pediatric area. However, research on 3MST with children and adolescents is still rare, which makes it difficult to use it in this group. The need for research on the psychometric properties of functional tests is evident to promote safety and credibility of these outcomes when assessing the functional status and clinical evolution of pediatric patients.

## CONCLUSION

Evidence on reproducibility and reliability for 6MWT and ISWT are good, but moderate for 3MST. ISWT was proven to have high validity measures for healthy children and children with chronic respiratory diseases. Measures of validity for 6MWT vary widely across populations studied and should consider each disease’s condition. The validity of 3MST has yet to be clarified, and further studies in the pediatric population are needed. Future research should explore the ability of such tests to measure significant and clinically important changes in different groups of children with cardiorespiratory diseases.
